# Current Advances in the Application of Raman Spectroscopy for Molecular Diagnosis of Cervical Cancer

**DOI:** 10.1155/2015/561242

**Published:** 2015-06-09

**Authors:** Inês Raquel Martins Ramos, Alison Malkin, Fiona Mary Lyng

**Affiliations:** ^1^DIT Centre for Radiation and Environmental Science, Focas Research Institute, Dublin Institute of Technology, Kevin Street, Dublin 8, Ireland; ^2^School of Physics, Dublin Institute of Technology, Kevin Street, Dublin 8, Ireland; ^3^School of Biological Sciences, Dublin Institute of Technology, . Kevin Street, Dublin 8, Ireland

## Abstract

Raman spectroscopy provides a unique biochemical fingerprint capable of identifying and characterizing the structure of molecules, cells, and tissues. In cervical cancer, it is acknowledged as a promising biochemical tool due to its ability to detect premalignancy and early malignancy stages. This review summarizes the key research in the area and the evidence compiled is very encouraging for ongoing and further research. In addition to the diagnostic potential, promising results for HPV detection and monitoring treatment response suggest more than just a diagnosis prospective. A greater body of evidence is however necessary before Raman spectroscopy is fully validated for clinical use and larger comprehensive studies are required to fully establish the role of Raman spectroscopy in the molecular diagnostics of cervical cancer.

## 1. Raman Spectroscopy—What Is It and How Does It Work?

**Figure 1:**
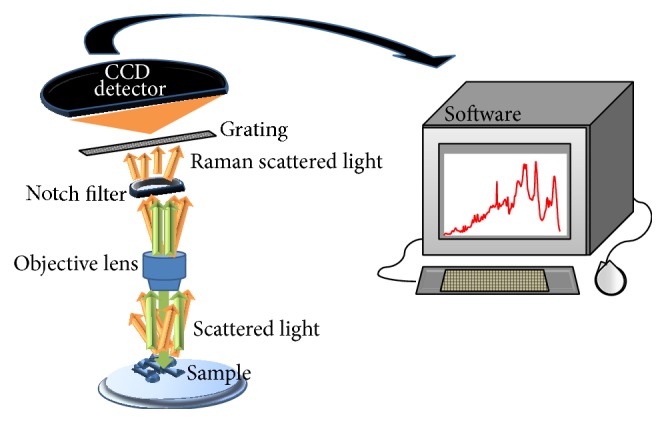
Schematic showing the process involved in Raman spectra collection. When the sample is illuminated by an incident monochromatic light, the majority of the scattered light is of the same wavelength—elastically scattered (green arrow). A notch filter is therefore used to block the elastically scattered light which would otherwise overwhelm the weak signal of the Raman or inelastically scattered light (orange arrow). The Raman scattered light may be dispersed according to wavelength through a grating and detected by a CCD (charge-coupled device) detector. A Raman spectrum is finally shown upon software analysis.

The physical phenomenon of Raman scattering, also known as the Raman effect, has been extensively studied since it was first discovered in 1928 by the Indian physicist C. V. Raman. It works on the principle that a small fraction (approximately 1 in 10 million) of the radiation scattered by certain molecules differs from that of the incident beam, and that the shift in wavelength depends upon the chemical structure of the molecules responsible for the scattering [1]. Raman spectra are acquired by irradiating a sample with a powerful laser source of usually visible or near-infrared monochromatic radiation and measuring the scattered radiation with a suitable spectrometer [1, 2].  Figure 1 shows the process involved in collection of Raman spectra.

**Figure 2:**
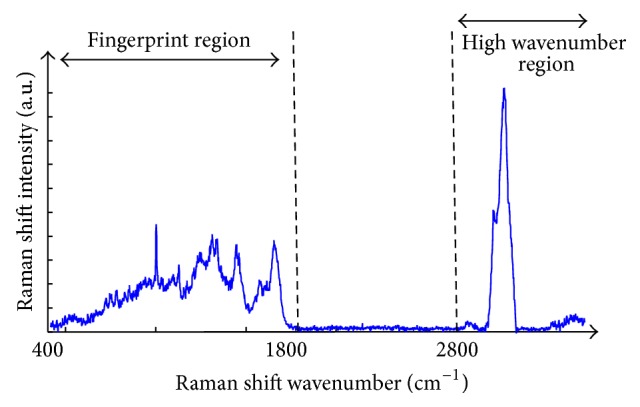
Raman spectrum of cervical cancer CaSki cell line. The variation of Raman shift wavelength is expressed in wavenumbers (cm^−1^) and can be observed along the *X*-axis whilst the intensity is represented along the *Y*-axis. The fingerprint and the high wavenumber (HW) regions of the spectrum are indicated by the arrows.

**Figure 3:**
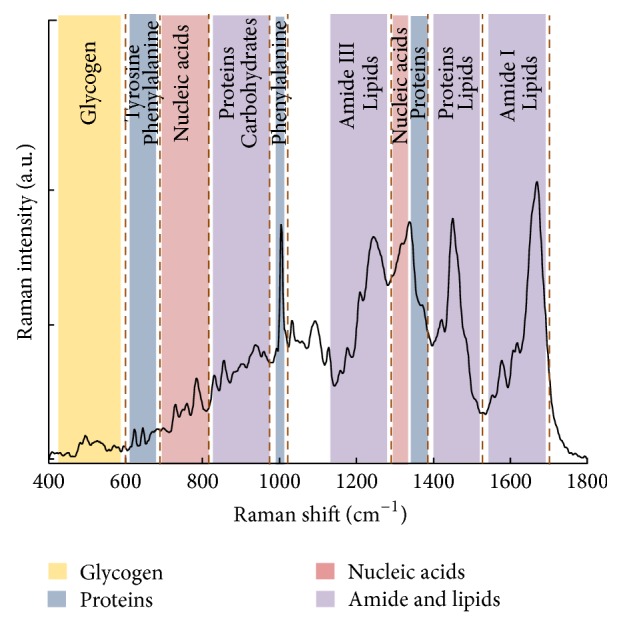
Fingerprint region of the Raman spectrum of cervical cancer CaSki cell line. The major assignments related to glycogen, proteins, lipids, and nucleic acids are highlighted.

Knowing the frequency of the incident light and measuring the frequency of the Raman scattered light, it is possible to calculate the vibrational energy difference. This energy is known as the Raman shift and is usually expressed in wavenumbers (cm^−1^) in a plot known as the Raman spectrum. Raman spectral features can be used as identification markers of particular substances because complex molecules have several specific vibrational energy modes allowing the Raman spectrum of each substance to be highly specific and distinctive [3].  Figure 2 shows an example of a Raman spectrum recorded from a cervical cancer cell line, CaSki. The full spectral range is shown from 400 to 3500 cm^−1^, including the fingerprint region, 400 to 1800 cm^−1^, and the high wavenumber (HW) region, 2800 to 3500 cm^−1^.  Figure 3 shows the fingerprint region in more detail with the major assignments related to glycogen, proteins, lipids, and nucleic acids highlighted.

Raman spectroscopy has been applied in numerous scientific fields, from chemistry and biochemistry to arts and archaeology, as a powerful spectroscopic technique which allows a spectral fingerprint capable of identifying the structure and function of molecules, cells, tissues, or materials [4, 5]. In particular, its application to medical diagnostics has been of increasing interest in the past few decades [6].

Raman spectroscopy has been reported for the detection of different types of pathologies, including cancer [4, 6–11]. A large number of studies concerning the investigation of cervical cancer with this particular vibrational spectroscopic technique have demonstrated its usefulness in understanding the disease progression at the molecular level. This review aims to compile the most significant achievements in this emerging research area. Methodologically, PubMed, Web of Science, and publicly available websites were searched for original data and literature in English using the following keyword combination: cervical cancer and Raman spectroscopy.

## 2. Cervical Cancer

Cervical cancer refers to any malignant neoplasm arising from the uteri* cervix*. As the fourth most common cancer in women worldwide and the fourth leading cause of female cancer deaths, cervical cancer is a key research area [12]. Its most common onset site is the cellular junction or transformation zone, where the stratified squamous epithelium of the* ectocervix* meets the columnar mucus-secreting epithelium of the* endocervix*. The most frequent types of cervical cancer are thus squamous cell carcinoma (SCC) and adenocarcinoma (ADC) [13, 14].

Persistent Human Papilloma Virus (HPV) infection is accepted as the leading aetiological agent for cervical cancer [15]. HPV is a circular double-strand DNA virus of almost 8000 bp belonging to the Papillomaviridae family. From more than 150 different genotypes, only 40 are reported to infect the anogenital tract, typically classified as high- or low-risk according to their ability to cause a recurrent infection [15, 16]. After HPV infection, dysplasia usually develops in the transformation zone. Low grade dysplasia can spontaneously regress without leading to cervical cancer [13, 17]. However some lesions progress to moderate and subsequently severe dysplasia, finally progressing to invasive cancer. For this reason, cervical cancer is postulated as a progressive disease [13, 17].

The implementation of coordinated and organized cytology screening programmes in developed countries has resulted in a marked decrease of the disease over the past decades; however cervical cancer is still a major problem in developing countries where approximately 80% of the cases occur [18]. The existing screening programmes are based on the microscopic evaluation of liquid based cytology and despite a high specificity of 95 to 98%, sensitivity varies from 74 to 96% [19, 20]. For this reason, other methods such as automated cytology and HPV testing have been studied in an attempt to reduce false negative rates.

An abnormal Pap smear is usually followed by colposcopy, biopsy, and histological confirmation of the diagnosis. Despite its slowness the major concerns about this procedure are the subjectivity of the grading characteristics and the fact that premalignancy or early malignancy stages could be missed due to their low morphologic perceptibility. Alongside other spectroscopy techniques such as FTIR (Fourier Transform Infrared) [21–23] and fluorescence spectroscopy [24–26], Raman spectroscopy has, in recent years, been acknowledged as a promising biomedical tool.

## 3. Raman Spectroscopy for Cervical Cancer

Table 1 compiles all the Raman spectroscopy studies concerning cervical cancer reported in the literature until September 2014 and discussed in this review. For clarity purposes it is important to explain a few terms that will be recurrent throughout the review.* In vivo* measurements relate to those acquired directly from the cervix of patients,* ex vivo* refers to the measurements acquired from the surface of biopsies and other surgical material extracted from the patients' cervix, and* in vitro* refers to spectra obtained from cell lines. Formalin fixed paraffin preserved (FFPP) histological sections and cytology samples are referred to separately.

### 3.1. *In Vivo* Spectra Recorded from the Patient

Mahadevan-Jansen et al. in 1998 were the first to show the potential of Near Infrared (NIR) Raman spectroscopy to detect cervical precancers amongst other pathologies. They developed a compact fibre-optic probe which they used to record* ex vivo* and* in vivo *spectra [27, 28].

The overall* ex vivo* conclusions stated that, in the Raman spectrum of squamous intraepithelial lesions, peaks attributed to collagen (1656, 1070 cm^−1^) consistently decreased in intensity while peaks assigned to phospholipids, DNA, and glucose 1-phosphate (1454, 1330, and 978 cm^−1^) increased in intensity. These findings were attributed with tumour progression, as the number of cells in the epithelium increases with lesion development. Furthermore, multivariate statistical analysis allowed the differentiation of precancers from all other tissues with sensitivity and specificity rates of 82% and 92%, respectively [28]. Their exploratory* in vivo* results showed broadly similar Raman spectra at the fingerprint region [27]. The main differences were (1) a band at 936 cm^−1^ only observed* in vivo*, (2) a peak at 978 cm^−1^ that was not consistently observed in* ex vivo* spectra, and (3) an amide band at 1252 cm^−1^ that was more prominent in the* in vivo *spectra. The authors highlighted the need to increase patient numbers so the* in vivo* technology could be clinically relevant [28].

Advances in fiber-optic technology led Utzinger et al. to further assess the viability of Raman spectroscopy to detect and classify cervical precancer lesions [29]. In a small clinical trial it was concluded that Raman spectra acquired from* in vivo* sampling were comparable with histopathology reports. The results showed increased Raman intensity of phospholipids and DNA assignments, ~1330, 1454, and 1650 cm^−1^, respectively, as the lesions progressed to high-grade dysplasia [29]. Despite these encouraging results, the authors noted the heterogeneity of the tissue and thus the possible contribution of normal epithelial cells to the spectral data. It was also suggested that further technological advances were once again needed to assess performance in large scale clinical trials [29].

The same group have investigated the influence of hormonal changes, particularly menstrual cycle and menopausal state, and by introducing these into the* in vivo* diagnosis algorithm, Kanter et al. improved the overall accuracy of Raman spectroscopy to 94% [30] reaching 97% for low-grade dysplasia detection [31]. Postmenopausal, perimenopausal, and premenopausal normal cervix before and after ovulation showed subtle but consistent differences at 1250 cm^−1^ and 1300–1320 cm^−1^, assigned to collagen and other cellular features like lipids, Amide III, and nucleotides [31]. Similarly, previous disease history and the proximity to malignant lesions were also shown to influence Raman spectral profiles. The principal qualitative differences between “true” normal and “previous disease” normal spectra were found in the 1200–1400 cm^−1^ range where assignments to proteins and collagen type I were higher in “true” normal spectra whilst the DNA and glycogen assignments (~1330 cm^−1^) were higher in “previous disease” normal Raman spectra [32]. The same range was also found to comprise the most significant differences between Raman spectra of “true” normal, “adjacent to disease” normal, and low- and high-grade dysplasia. Collagen assignment was again higher in both “true” and “adjacent to disease” normal spectra whereas DNA was higher in low and high grade dysplasia spectra [32].

In an attempt to further establish the greatest sources of intraclass variation among normal Raman spectra, Vargis et al. investigated race and ethnicity, body mass index (BMI), parity, and socioeconomic status in their* in vivo* study. The results showed that only BMI and parity were significant sources of variation within normal spectra. Their influence on dysplasia and disease remains to be assessed [33].

### 3.2. *Ex Vivo *Spectra Recorded from Excised Patient Tissue

Krishna et al. reported Raman spectral differences between normal and malignant biopsy samples. Amides I and III and structural proteins such as collagen seemed to be characteristic of normal tissue whilst DNA, lipids, and noncollagenous proteins dominated the abnormal spectral features [34]. Keller et al. showed that Raman spectral profiles from the stroma below epithelium with HPV associated histological changes had differences in DNA (1316 and 1334 cm^−1^) and glycogen (1048, 1083, 1256, and 1333 cm^−1^) assignments [35]. Further differences at 1260 and 1304 cm^−1^ Amide III band were proposed to be related with the angiogenesis process or to the fact that disease may have extended without visible histologically effects [35]. While increased DNA levels and decreased glycogen levels as dysplasia progresses had been described before, this was the first report of alterations of the histologically normal stroma below diseased epithelium. Further study is therefore warranted as disease classification depends on stromal invasion.

The role of cervicitis in Raman spectroscopy diagnosis of low-grade dysplasia was investigated by da Silva Martinho et al. Despite an overall sensitivity and specificity of 93% and 85%, the results showed that spectral changes observed at 857, 925, ~1247, 1370, and 1525 cm^−1^ vibrational bands resulted in the cervicitis group falling mid-way between the normal and low-grade dysplasia groups. The data showed that a severe inflammatory condition such as cervicitis makes the identification and correct diagnosis of early malignancy stages such as low-grade dysplasia difficult and must therefore be taken into account when developing data analysis algorithms [36].

Finally NIR micro-Raman spectroscopy study by Kamemoto et al. showed that Raman spectra from collagen bands at the low frequency 775–975 cm^−1^ region distinguish normal from cervical cancer cells, and that this is concordant with the analysis of C–H stretching in high wavenumber region (2800–3700 cm^−1^) [37].

### 3.3. FFPP Sections Spectra Recorded from Histological Sections

Archival FFPP material is extremely valuable as it allows retrospective studies to be undertaken after diagnosis and outcome is known. Raman spectroscopy studies have been undertaken on histological FFPP sections further confirming Raman spectroscopy as a powerful informative tool in cervical cancer research.

Krishna et al. studied formalin fixed cervical tissues by both Raman and FTIR spectroscopy, reporting the discrimination of malignant tissues through both techniques. In Raman spectra, differences in protein, lipids, and nucleic acid peaks were observed along with stronger Amide III assignments which is supportive of disordered, helical secondary structure of protein components in malignant conditions [38].

Further confirmation of the potential of Raman spectroscopy for cervical cancer was reported by Lyng et al. who demonstrated the viability of using FFPP samples and investigated the underlying biochemical changes associated with cervical precancer and cancer [39]. Results showed a reduction in glycogen bands at 482, 849, and 938 cm^−1^ and an increase in nucleic acid bands at 724, 779, 829, 852, 1002, 1098, 1240, and 1578 cm^−1^ in cervical precancer and cancer. An increase intensity of Amide I was also reported [39].

### 3.4. *In Vitro *Spectra Recorded from Cell Lines

Yazdi et al. described the use of UV resonance Raman spectroscopy at 257 nm to distinguish between normal and malignant breast [MCF-10A, MCF-7 McGuire, and MDA-MB435] and cervical [CrEc-Ec 4665 (primary culture from normal cervix epithelium), SiHa, and HeLa] cultured cells. They reported an increase in DNA/protein ratio and a change in the purine scattering in malignant cells, suggesting the application of resonance Raman spectroscopy in cytology screening by monitoring DNA and RNA differences between normal and abnormal cells [40].

Despite being the main aetiological factor in cervical cancer, HPV was only investigated by Raman spectroscopy towards the end of the last decade with a cell culture study by Jess et al. [41]. Raman microspectroscopy was used to discriminate PHK (primary human keratinocytes), PHK E7 and CaSki cells, where PHK E7 cells express the E7 gene of HPV16 and CaSki expresses HPV16. The mean Raman spectra showed variations at DNA and protein level, consistent with HPV gene expression and malignancy in both live and fixed cells. Together with principal component analysis (PCA) results, Raman spectroscopy was shown to be a valuable tool in identifying and characterizing the different stages of HPV-associated malignancies [41].

Ostrowska et al. applied both FTIR and Raman spectroscopy to the study of cervical cancer cell lines. Their data suggest that HPV negative (C33a) and low HPV copy number (SiHa with 1-2 copies) cell lines are biochemically very similar but significantly different from mid (HeLa) and high (CaSki) HPV copy number cell lines. The main variations were observed for protein, nucleic acid, and lipid levels and were confirmed by both mean spectra and PCA analysis [42]. Discrimination of the cell lines based on HPV integration shows the potential of Raman spectroscopy to identify HPV induced biochemical changes [42].

Worthy of highlight is also a comparative study by Kim et al. [43] of the distribution of intracellular components in cells expressing HPV16 E6 oncoprotein. The key finding of this Raman mapping study is that E6 oncoprotein expression induces major phenotypic changes in the cells which are also targeted by an HIV antiviral drug—Indinavir [43].

Vargis et al. [44] also showed Raman microspectroscopy to successfully detect HPV and differentiate specific virus strains, in a complementary cell line and* in vivo* study with cellular pellets from cytology samples. Normal HPV negative cell line NHEK was used alongside three cervical carcinoma cell lines: HPV positive (HeLa and SiHa) and HPV negative C33a. Specificity values of 89–97% for cell lines and 98.5% for cytology samples are extremely encouraging and confirm the enormous potential of Raman spectroscopy to provide an accurate differential diagnosis [44].

### 3.5. Cytology Spectra Recorded from Exfoliated Cells

Rubina et al. used Raman spectroscopy to distinguish between 49 cervical cancer and 45 negative control cytology samples. Cellular pellets were generated from ThinPrep material and subjected to Raman analysis. Amide I (1660 cm^−1^), *∂*CH_2_ (1450 cm^−1^), and phenylalanine (1002 cm^−1^) were the main features dominating the control Raman spectra whereas the spectra of cervical cancer samples were dominated by blood features such as fibrin (1570 cm^−1^) and heme (1620 cm^−1^). PCA-LDA (linear discriminant analysis) showed a classification efficiency of ~90% but the loadings were suggestive of blood as the major discriminative factor between the two groups. As bleeding is a common occurrence in cervical infections, uterine cancer, and menstrual cycle, 57 samples (28 controls and 29 cancers) were further treated with red blood cell lysis buffer prior to Raman acquisition. The absence of heme and fibrin bands confirmed the effective removal of blood from the samples and an increase in protein content (at 1006, 1450, and 1660 cm^−1^) and change in their secondary structure due to positive Amide III bands was observed. In this case the PCA-LDA analysis showed a classification efficiency of ~80%. Sample heterogeneity and the fact that the distribution of the abnormal cells in the cervical cancer specimens can vary from 1-2% to 20–40% were suggested as the major causes of misclassification. The authors suggested further studies on pure cancerous and precancerous specimens as a means to build standard and validation models that could then be applied to blinded specimens [45].

### 3.6. Treatment Response

In their dual Raman and FTIR study, already mentioned in the FFPP section, Krishna et al. also presented data concerning Raman spectra after radiotherapy cycles, showing small changes, especially in antioxidant levels [38]. In a further* ex vivo* pilot study to detect radiotherapy response [46], tissues were collected after a second fraction of radiotherapy and classified based on clinical evaluation into complete, partial, and no response. Raman spectra were acquired and PCA provided a clear separation between responding and nonresponding samples as well as between complete and partial radiotherapy response.

In a more recent* ex vivo* study, Rubina et al. explored the feasibility of fibre-optic-based Raman spectroscopy in predicting tumour response to concurrent chemoradiotherapy. Their PCA classification pattern also showed encouraging results despite the need of a greater body of evidence [47].

A study by Duraipandian et al. used HW Raman spectroscopy to noninvasively assess,* in vivo*, the effect of Vagifem (oestrogen therapy) treatment in women [48]. A bimolecular Raman spectroscopy model could not only successfully identify hormone/menopausal related changes in cervical epithelium, but also assess the effect of Vagifem treatment during colposcopic inspections as the protein and lipid Raman signals increase after treatment and start to resemble premenopausal values [48].

### 3.7. Improving Data Analysis and Recording

Improving overall sensitivity and specificity of Raman spectroscopy for* in vivo* diagnosis of cervical cancer has also led researchers to address better algorithms and methods for statistical analysis. A study by Kanter et al. explored binary and multiclass discrimination algorithms to analyse Raman spectroscopy data: maximum representation and discrimination feature (MRDF) and sparse multinomial logistic regression (SMLR). Although both algorithms provided an improvement over the current method of diagnosis, colposcopy-guided biopsy (with sensitivity of 87% and specificity of 72%), the use of a multiclass algorithm improved the overall Raman spectroscopy sensitivity from 92% to 98% and the specificity from 81% to 96% [49].

Similarly, Duraipandian et al. investigated the application of genetic algorithm-partial least squares-discriminant analysis (GA-PLS-DA) with double cross-validation (dCV). By employing a GA-PLS-DA algorithm which used significant Raman bands selected from 925–935, 979–999, 1080–1090, 1240–1460, 1320–1340, 1400–1420, and 1625–1645 cm^−1^, a 72.5% specificity and 89.2% sensitivity for precancer detection were achieved and could therefore be further investigated as a feasible alternative to current PCA methods [50].

Still in the* in vivo* context, modifications in the recording process have also been considered and reported in the literature. HW Raman spectroscopy, 2800–3700 cm^−1^, was successfully described by Mo et al. with 93.5% and 97.8% diagnostic sensitivity and specificity, respectively [51]. The results showed that the intensity of the Raman signal within the 2800–3035 cm^−1^ range, which comprises proteins and lipids, from dysplastic tissue, was significantly lower than that observed for normal tissue. An increase in the vibrational signal of water from the dysplastic tissue was also observed and in line with that reported by FTIR spectroscopy [10, 11]. The authors further supported these observations with literature concerning the increase of aquaporins at the dysplastic cell membrane and the fact that higher DNA levels or hydration of DNA due to the unfolding step in cell division could also account for this observation [51].

Simultaneous fingerprint and HW Raman spectroscopy have also been described by Duraipandian et al. who showed their complementary potential and ability to improve early disease detection. The sensitivity and specificity values of 85% and 81.7%, respectively, for integrated fingerprint and HW Raman spectroscopy were shown to be higher than those of fingerprint or HW Raman spectroscopy alone [52].

### 3.8. Future Perspectives

An exploratory work on surface-enhanced Raman spectroscopy (SERS) for cervical cancer diagnosis through blood plasma analysis was recently reported by Feng et al. Comparing blood plasma samples from clinically and histopathologically confirmed healthy volunteers and cervical cancer patients, results showed that PCA-LDA algorithms yielded better sensitivity (96.7%) and specificity (92%) than empirical algorithms based only on the integration area of SERS spectral bands of 1310–1430 and 1560–1700 cm^−1^ [53].

Along the same lines, González-Solís et al. published a study on cervical cancer detection based on Raman spectroscopy of serum samples. The study reported higher levels of carotenoids and protein components in control samples whereas assignments to glutathione and tryptophan were more intense in the spectra of abnormal samples. Despite a small number of patient samples (3 CIN I and 19 SCC), PCA analysis yielded a sensitivity of 100% and a specificity of 97.1% [54].

## 4. Summary

All pathologies are marked by fundamental biochemical changes at the molecular, cellular, and tissue level. The identification and further understanding of these changes would allow improved diagnosis and treatments, as well as overall management and disease survival. The potential of Raman spectroscopy in molecular diagnostics relies on its ability to determine and characterize the unique fingerprint of a sample at the biochemical level.

The high incidence rate of cervical cancer as well as its progressiveness has led researchers to continually examine and pursue better diagnosis, prognosis, and treatment techniques to decrease mortality rates and comorbidity from the disease. As highlighted, not only does Raman spectroscopy have the potential to identify cancerous and precancerous tissue, it also has the ability to probe deeper into the disease fingerprint to elucidate its underlying mechanisms. By implementing variations of the technique to study a wide range of samples such as commercially available cell lines, FFPP sections, and* in vivo* and* ex vivo* tissue, the potential of Raman spectroscopy as a viable option for a future diagnostic technology of cervical cancer and other disease states has been shown. In cervical cancer, a number of different factors including HPV infection, hormonal imbalances, and inflammatory infection have already been reported to influence Raman spectra. Whilst these could be seen as limitations, they actually prove the sensitivity of the technique and support additional evidence generated by other approaches such as proteomics and virology.

However, lack of information regarding which data was considered for the sensitivity and specificity values reported, as well as the lack of positive and negative predictive values, calls for the standardization in the reporting of these important performance measurements. Likewise, sample handling and processing ought to be reported as it can influence Raman spectroscopy profiles. Finally, whilst acknowledging the exploratory nature of most studies and the difficulty in obtaining patient samples, a frank criticism is the small sample size of most reported studies. Although spectroscopically significant due to the high number of spectroscopic measurements, more samples are required to assess the biological and pathological relevance and reproducibility.

A far greater body of evidence is still required before this technology can make head way in a clinical setting. For instance, the engagement of the clinical community in supporting more comprehensive studies both* in vivo* and* ex vivo*, cognisant of all variables and considering a wide range of controls, gathered from a representative spectrum of the population would be vital to take the technique a step closer to cervical cancer diagnosis. If such studies could be undertaken and the reliability of the technique proven, Raman spectroscopy could have a real future in clinical diagnostics of cervical cancer and similar pathologies.

## Figures and Tables

**Table 1:** Raman spectroscopy studies concerning cervical cancer reported in the literature until September 2014 sorted by diagnosis (D), treatment response (R), and further conditions analysed. Sampling numbers and data analysis methodology are also indicated as maximum representation and discrimination feature (MRDF), sparse multinomial logistic regression (SMLR), principal component analysis (PCA), linear discriminant analysis (LDA), genetic algorithm-partial least squares-discriminant analysis (GA-PLS-DA), partial least squares-discriminant analysis (PLS-DA), Fisher's discriminant analysis (FDA), principal component analysis logistic regression (PCA-LR), and spectral analysis when no multivariate statistical method was reported.

Sampling type	Sampling numbers	Year	Authors (research group)	Raman spectroscopy [spectral region; laser used]	Sort category	Data analysis methodology	Other considerations
*In vivo n* = 11	Not disclosed	1998	Mahadevan-Jansen et al. [28]	Fingerprint region; 789 nm	D	Spectral Analysis	—
	25	2001	Utzinger et al. [29] (Mahadevan-Jansen group)	1000–1800 cm^−1^; 789 nm	D	Spectral analysis	—
	66	2009	Kanter et al. [30] (Mahadevan-Jansen group)	Fingerprint region; 785 nm	D	MRDF and SMLR	Multiclass development
	31	2009	Kanter et al. [31] (Mahadevan-Jansen group)	Fingerprint region; 785 nm	D	MRDF and SMLR	Hormonal variation influence
	46	2009	Mo et al. [51] (Huang group)	HW (2800–3700 cm^−1^) region; 785 nm	D	PCA-LDA	—
	102	2009	Kanter et al. [49] (Mahadevan-Jansen group)	Fingerprint region; 785 nm	D	MRDF and SMLR	—
	172	2011	Vargis et al. [32] (Mahadevan-Jansen group)	Fingerprint region; 785 nm	D	SMLR	Normal variability and previous disease
	29	2011	Duraipandian et al. [50] (Huang group)	Fingerprint region; 785 nm	D	GA-PLS-DA	Additional genetic algorithm techniques
	75	2011	Vargis et al. [33] (Mahadevan-Jansen group)	Fingerprint region; 785 nm	D	MRDF and SMLR	Investigation of normal patient variability
	44	2012	Duraipandian et al. [52]	Fingerprint & HW (2800–3700 cm^−1^) region; 785 nm	D	PLS-DA	—
	26	2013	Duraipandian et al. [48] (Huang group)	HW (2800–3700 cm^−1^) region; 785 nm	—	PLS-DA	Vagifem treatment

*Ex vivo n* = 7	20	1998	Mahadevan-Jansen et al. [27]	Fingerprint region; 789 nm	D	FDA and PCA	—
	150	2006	Krishna et al. [34]	Fingerprint region; 785 nm	D	PCA	—
	66	2008	Vidyasagar et al. [46] (Krishna group)	Fingerprint region; 785 nm	R	PCA	—
	102	2008	Keller et al. [35] (Mahadevan-Jansen group)	Fingerprint region; 785 nm	D	MRDF and SMLR	Investigation of temporal and spatial effects
	63	2008	da Silva Martinho et al. [36]	Fingerprint region; 1064 nm	D	PCA-LR	Cervicitis influence
	14	2010	Kamemoto et al. [37]	Fingerprint region; 785 nm	D	Spectral analysis	—
	42	2013	Rubina et al. [47] (Krishna group)	Fingerprint region; 785 nm	R	PCA-LDA	Chemoradiotherapy

*In vitro n* = 5	—	1999	Yazdi et al. [40] (Richards-Kortum group)	600–2500 cm^−1^; resonance, 257 nm	D	Spectral analysis	—
	—	2007	Jess et al. [41] (Herrington group)	Fingerprint region; 785 nm	D	PCA	—
	—	2010	Ostrowska et al. [42] (Lyng group)	Fingerprint region; 532 nm	D	PCA	HPV influence
	—	2010	Kim et al. [43] (Goodacre group)	Fingerprint region; 830 nm	—	Spectral analysis	HPV16 influence (E6 protein)

*In vitro* and cytology	50	2012	Vargis et al. [44] (Mahadevan-Jansen group)	Fingerprint region; 785 nm	—	SMLR	HPV detection

Cytology *n* = 2	94	2013	Rubina et al. [45] (Krishna group)	Fingerprint region; 785 nm		PCA-LDA	—

FFPP *n* = 2	18	2007	Krishna et al. [38]	Fingerprint region; 785 nm	D	PCA	—
	60	2007	Lyng et al. [39]	Fingerprint; 514.5 nm	D	PCA-LDA	—

Blood *n* = 2							
Plasma	110	2013	Feng et al. [53] (Huang group)	350–1750 cm^−1^; SERS, 785 nm	D	PCA-LDA	—
Serum	42	2014	González-Solís et al. [54]	Fingerprint region; 830 nm	D	PCA	—
